# Building research capacity for evidence-informed tobacco control in Canada: a case description

**DOI:** 10.1186/1617-9625-5-12

**Published:** 2009-08-07

**Authors:** Paul W McDonald, Sarah Viehbeck, Sarah J Robinson, Scott T Leatherdale, Candace IJ Nykiforuk, Mari Alice Jolin

**Affiliations:** 1 Department of Health Studies and Gerontology, University of Waterloo Waterloo, Ontario, Canada; 2 Population Health Research Group, University of Waterloo Waterloo, Ontario, Canada; 3 McMaster Child Health Research Institute, McMaster University, Hamilton, Ontario, Canada; 4 Department of Population Studies and Surveillance, Cancer Care Ontario, Canada; 5 Dalla Lana School of Public Health, University of Toronto, Canada; 6 Centre for Health Promotion Studies, University of Alberta, Canada

## Abstract

Tobacco use remains the leading cause of death and disability in Canada. Insufficient research capacity can inhibit evidence-informed decision making for tobacco control. This paper outlines a Canadian project to build research capacity, defined as a community's ability to produce research that adequately informs practice, policy, and future research in a timely, practical manner. A key component is that individuals and teams within the community must mutually engage around common, collectively negotiated goals to address specific practices, policies or programs of research. An organizing framework, a set of activities to build strategic recruitment, productivity tools, and procedures for enhancing social capital are described. Actions are intended to facilitate better alignment between research and the priorities of policy developers and service providers, enhance the external validity of the work performed, and reduce the time required to inform policy and practice.

## Introduction

Early tobacco control interventions were heavily influenced by research evidence demonstrating the health burden associated with tobacco use. For example, the 1962 report by the Royal College of Physicians and Surgeons in the United Kingdom prompted countries such as Sweden to dedicate large sums of government funding for public education campaigns [1]. The 1964 United States Surgeon General Report provided a similar foundation for tobacco control in North America [2]. More recently, comprehensive scientific summaries have been used to inform the development of clinical practice guidelines for smoking cessation in several countries, as well the World Health Organization's Framework Convention on Tobacco Control [3].

Despite significant progress even relatively aggressive, sustained strategies such as the tobacco control program in California [4] have failed to reduce the prevalence of adult tobacco use to below 12 percent. Moreover, the characteristics and behaviours of smokers today are substantially different from the characteristics and behaviours of the smokers on which current interventions were tested. As such, continued reductions in tobacco use and its associated health and economic burden will require the development and successful implementation of new strategies. Having sufficient numbers and adequate distribution of well trained, innovative and knowledgeable researchers who value practice- and/or policy-relevant research will be of paramount importance for achieving this aim.

Evidence-informed policies and programs can easily be impeded when there is insufficient capacity to produce new and/or relevant research to meet the information needs of decision makers in a timely manner. For example, the Canadian Cancer Society (CCS), a non-profit organization dedicated to cancer control, demonstrated this lack of alignment between research and the needs of public health practitioners and policy makers. The CCS commissioned a research team to answer four basic questions: 1) are group counseling programs for smoking cessation effective? 2) if so, what is the optimal content of the sessions? 3) what is the optimum number and frequency of sessions that should be offered? and, 4) what are the characteristics of the most effective facilitators? A comprehensive literature review analyzed 40 years of published and unpublished studies and concluded that due to consistent deficiencies in purpose, design and reporting, available research could only address the first question; that group programs for smoking cessation are effective [5]. Hence, even in a relatively established field such as smoking cessation treatment, research is not always able to inform the operational issues of greatest salience to decision makers.

Capacity for innovation can be limited when there is too much disciplinary or geographic homogeneity among researchers. For example, one discipline may be in a position to conduct research on whether group treatments for smoking cessation are effective, but not have the background to properly vary treatment content, delivery modes, cost efficiency, or cost effectiveness. Research capacity is often concentrated within a handful of institutions in a limited number of geographic regions or countries [6]. Too much homogeneity may limit the internal and external validity of research [7]. Homogeneity also makes it more challenging to develop or enhance relationships between research producers and the national, state and/or local program providers/policy developers who are crucial for facilitating the integration of research into practice and, vice versa.

This paper outlines an approach being used in Canada to create research capacity in the field of tobacco control. We describe the guiding constructs of our approach, our project framework, and some processes and challenges associated with building a pan-Canadian community.

## Context for the Canadian Project

The desire to justify and implement comprehensive, well funded, evidence-based tobacco control strategies by public and non-profit organizations has rapidly increased the demand for research and evaluation capacity [8,9]. However, until recently, relatively few Canadian researchers focused their efforts on tobacco control.

In order to facilitate evidence based tobacco control, leading public and non-profit research funding bodies in Canada took three steps. First, in 1997 the National Cancer Institute of Canada (NCIC) lead the creation of the Canadian Tobacco Control Research Initiative (CTCRI) to strategically direct research funding to priority areas for tobacco control (cf.  for more information). Second, in 2002 the Canadian Institutes of Health Research (CIHR) began funding two projects to enhance the training of tobacco control researchers in Canada through the Strategic Training Program in Tobacco Research (cf.  and ). These two training programs shared the goal of funding graduate studentships, post doctoral awards, and research fellowships. Third, CTCRI and its partners at the CIHR, the NCIC, the Heart and Stroke Foundation of Canada, and Health Canada issued a request for applications to build interdisciplinary research capacity for tobacco research in Canada. Three successful Interdisciplinary Capacity Enhancement (ICE) teams each received $300,000 CDN a year for five years.

This paper outlines the rationale and approach that has been used by one of the ICE teams, the "Pan-Canadian Resource Network for Tobacco Control Research, Policy and Practice" (herein referred to as the ICE-PRN).

## Defining Research Capacity

**Figure 1:**
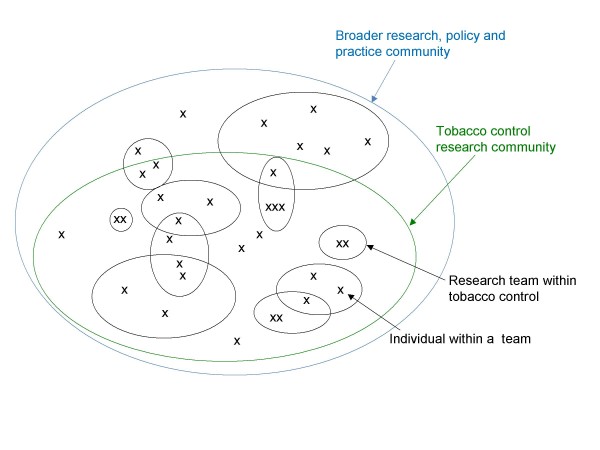
A representation of nested teams in tobacco control research.

The ICE-PRN began by defining research capacity as a research community's ability to produce research that adequately informs practice, policy, and future research in a timely, practical manner. In this paper, the "research community" refers to individuals affiliated with the ICE-PRN (through events or network activities), while the "tobacco control community" reflects the broader national (and international) community (cf Figure 1). Our project went beyond simply wanting to increase the number of researchers who direct their attention toward a problem and the number of publications they have. Rather, within our framework, capacity has also been regarded as a function of *who *actively participates in the community (diversity), *how *they work together (social capital), *what *tools they have (resources), *what *they work on (priority setting), and *how *effectively products are evaluated and shared with each other and those external to the research community (research and knowledge exchange). Therefore, as we will describe later, the process for enhancing research capacity has been regarded as equally important as the products of research that were put forth by the research community (including papers in peer reviewed journals, policy briefs, technical papers, presentations to practitioners and policy developers, assessment devices, monitoring tools, treatment and decision aids, etc.).

### Research Communities

Our notion of a "research community" paralleled Wenger's idea of a "Community of Practice" [10]. Our project attempted to facilitate the development of a research community made up of individuals and teams of people who mutually engaged around common, collectively negotiated goal(s) to address a specific practice, policy or program of research. Relations within teams were as important as relations across teams. Membership on teams frequently overlapped (Figure 1), allowing specific (often scarce) expertise to be shared efficiently and improving connectivity across teams. In a similar way, as illustrated in Figure 1, the tobacco control research community was nested within or intersected with other communities dealing with even more diverse problems, such as reducing chronic disease, which in turn may intersect with even larger fields such as public health.

For our purposes, communities and teams were dynamic structures that not only generated new knowledge, but helped place this knowledge in context and facilitated learning through member interaction. Communities and teams had specific boundaries, although membership was not assigned or based on whether or not an individual paid dues or was part of some formal register. Instead, community membership was centered around a mutual desire to stimulate learning and/or enhance decision making through research, mentoring and/or exchange with respect to a common problem (e.g., enhancing the salience of tobacco warning labels), practice (e.g., improving the effectiveness of telephone quitlines), or policy (e.g., eliminating point of purchase advertising of tobacco products). To be a community, individuals and teams required structure beyond the loose, informal, and sometimes chaotic interactions of traditional networks. In our context a community was defined by teams' and individuals' common and collective concerns about using research to inform decision making for tobacco control. Where communities may have had a broad mandate, teams within communities had a much more specific focus, such as conducting tobacco control research in school settings, or studying the impact of poverty on tobacco use.

### Social Capital

Kawachi and Kennedy [11] describe social capital as "the resources available to an individual through their social affiliations and membership in community organizations (p 173)". A common theme across commentators is that social capital enhances capacity by: i) facilitating innovation; ii) enhancing timely access for community members to information and expertise; iii) helping members to gain access to external gatekeepers, influencers, and key decision makers and stakeholders; and iv) assisting community members to put information in context. Not only is such an environment likely to be an incentive for attracting prospective community members, but it also improves the productivity of existing members whereby the achievement of certain ends would not be possible without the support of such an environment [12].

We adapted a working definition of social capital from Daniel and colleagues [13]. Accordingly, we regarded social capital as those "common social resources [within a research community] that facilitated information exchange, knowledge sharing, and knowledge construction through interaction, built on trust, [reciprocation, mutual norms and values], and maintained through shared understanding" (p. 2).

While factors within a community such as network configuration and connectivity were important determinants of whether knowledge was constructed and exchanged in a manner that produces a desired impact, so too were elements such as the level of trust and cohesion that existed between members, their willingness to reciprocate by both giving and receiving assistance, adherence to shared norms and values, and the creation and/or use of shared language and standards during interaction [12]. The teams and the community as a whole had to collectively generate social capital in order for all members within teams and communities to receive mutual benefit.

### Research and Knowledge Exchange

Early models of research translation emphasized the unidirectional flow of knowledge from research producers to research users. In order to improve receptivity to research, more recent conceptualizations have emphasized the need for bi-directional knowledge transfer [14-16]. However, our framework took this a step further and emphasized the need for continuous bi-directional *exchange *between research producers and intended users by integrating them into the same community where they are regarded as partners with complementary sets of expertise. Hence, research teams not only consisted of scientists, but also the key decision makers and program providers. Such infrastructure allowed research problems and priorities to be mutually identified so that studies were designed and disseminated with continuous and explicit input from (and value for) both researchers and program/policy decision makers [17].

Within such a system, the relative level of input from different contributors varied across time. For example, in the same way that methodologists tend to provide more input during the design and interpretation phases of a research study, so it was that research users were more involved in defining the problem, selecting relevant study populations and outcome measures, training intervention agents, assisting with data collection, and interpreting results. Research producers were more likely to exercise leadership by applying specific expertise in recommending various research designs and measurement standards, training data collectors, managing the data, and conducting the analyses. Negotiation points also occurred around issues of project feasibility, timelines, deliverables, and dissemination of results.

### Diversity

Our framework assumed that diversity enhances creativity and capacity. This assumption was consistent with the broader movement toward multi- and inter-disciplinary research. Our framework aimed to create diversity across four dimensions: discipline, sector, geography, and experience. Our ultimate aim was to facilitate interdisciplinary research (or multi-disciplinary research at the very least), and to not only encourage exchange and integration across disciplines, but to expand beyond the disciplines that have traditionally been involved in tobacco control (e.g., psychology, medicine, nursing, pharmacology, epidemiology, bio-statistics, economics, toxicology, etc.) to include non-traditional disciplines (e.g., political science, computer science, accounting, law, marketing and geography). We actively encouraged teams to include a sectoral mix of research producers and users to make research findings more relevant to end users, i.e., research outcomes that are compelling to decision makers are more apt to facilitate the uptake of results into policy and practice. Emerging evidence suggests that research was also more likely to be used when decision makers view it as relevant to their population constituency and trusted its source (i.e., researcher or sponsoring organization) [18].

It is conceivable that research communities which involved teams with geographic diversity may enhance external validity and trust to local audiences. Decision makers may trust the views of researchers that are perceived to have a personal stake in "getting the outcomes right" (i.e., stakeholders who are going to be impacted by the outcomes within the region), and are familiar with the values and culture(s) of the region. Having team members from different geographic regions also facilitated contact with key local decision makers because of personal connections and social networks; factors which may also assist in creating data collection opportunities.

Finally, our framework regarded mentorship as a key element of building future research capacity. We aimed to create teams and a community that included a rich mix of student trainees, new investigators and practitioners, more experienced investigators and practitioners, and decision makers.

### Priority setting and timing

In order to address the health and economic burden associated with tobacco use in a timely fashion, there was a need to prioritize and focus on the problems that might have the greatest immediate impact. Although priorities can either be identified from a research perspective or a practitioner and/or decision maker perspective, they can be influenced by different considerations such as infrastructure, timing, and political will. For instance, even if an important research priority has been identified through a policy window, if the research infrastructure was not in place to respond in a timely manner (e.g., data collection systems, scientific leadership, etc.), it would have been difficult to produce the evidence required to inform decisions. One must also consider that even if the research could be performed in an adequate period of time, action may not have been taken on the priority if the research findings were inconsistent with the decision making context (due to public opinion, funding availability, political ideology, etc.). By linking research producers and users through collaborative teams, built through mutual interest and negotiation, the potential to prioritize efforts towards key problems could be fostered [7]. When this interactive, efficient and focused team system is replicated for numerous priority areas by numerous teams within the broader tobacco control community, there lays the possibility to have more efficient and immediate impact at the population-level.

### Resources for productivity and exchange

Having sufficient financial and human resources are undeniably important for conducting quality and timely research. While researchers often dwell on the need to obtain new resources, a second strategy was to use existing resources more productively or efficiently. While our project attempted to make decision makers aware of the need for new and continuing investments in tobacco control research, an effective argument was to demonstrate that current resources were being used as responsibly and efficiently as possible. Moreover, an effective incentive for recruiting and keeping research users and producers within our community was to assist them to be more productive. To these ends, our project created a series of productivity tools and resources (see below for examples) for our community members.

A series of informal consultations with potential research community members suggested that, other than operational grant funding, the most acute capacity-related needs focused on four basic themes: i) improved access to and exchange of proprietary data; ii) improved access to participants and populations of study for research studies, and/or potential collaborators (including students and prospective supervisors); iii) improved access and exchange of research results and products; and iv) assistance to build skills and new research methods. Since many datasets are proprietary in nature (i.e., owned by the investigator(s) who collected the data), it was difficult to provide data and associated meta-data to persons outside the original research team. Further, the original research team may not have the necessary resources to fill external requests for data access. Second, there was a need for assistance in accessing study participants or populations when a researcher was not familiar with the specific data collection considerations associated with the topical area (e.g., data collection issues for school-aged youth are dramatically different from data collection issues for adults). Since it can also be difficult to find individual study samples which have suitable variability and number of study participants for answering certain research questions, data linkage may be required. Third, assistance was required to locate unpublished research products such as presentations, reports, and policy briefs which are not often widely available or accessible to all members of the research community. Finally, there was a need for modest funds to facilitate face-to-face exchanges and learning opportunities. Community members required money to form teams, develop mutual goals and action plans, learn from one another, and start building social capital. This included quick access to funds for initial preparatory work to support grant applications or to assess feasibility such as pilot testing new methods, estimating required sample sizes, etc.

## Organizing Framework and Objectives

**Figure 2:**
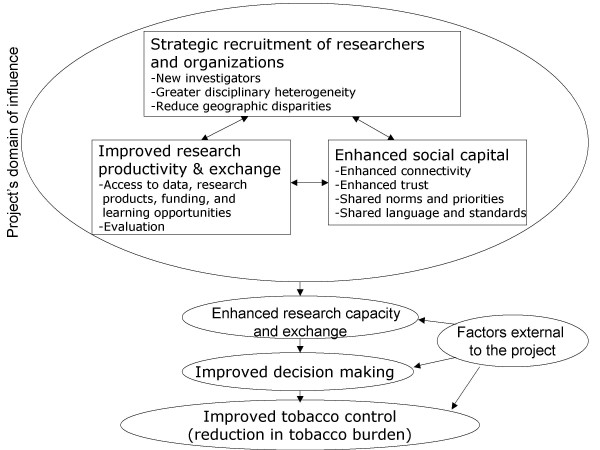
ICE-PRN organizing framework.

Figure 2 outlines the organizing framework and logic model for the project. The project goal was to build research capacity to produce rigorous, credible, and timely evidence which research users could draw upon to enhance their selection, allocation, and implementation of tobacco control resources, policies and programs. This, in turn, was intended to improve the ability of tobacco control initiatives to reduce the health and economic burden associated with tobacco use. The framework recognized that the ICE-PRN was only one source of influence toward this goal. Individuals, teams, and factors beyond our network were clearly important contributors.

Commensurate with the expressed needs of the community, our project logic aimed to enhance research capacity via three general mechanisms: strategic recruitment and training, building social capital, and creating tools for enhancing research productivity and exchange. Since each mechanism had an associated objective, we developed a complement of targeted activities to address these objectives. Our overall approach was informed by a comprehensive evaluation strategy intended to guide our progress and inform the broader literature on capacity building. Table 1 summarizes the relationships between activities and their associated objectives and themes. Table 2 summarizes core indicators for each objective.

**Table 1:** ICE themes, objectives, and associated activities

	THEMATIC AREAS				
	SOCIAL CAPITAL		STRATEGIC TRAINING & RECRUITMENT		RESEARCH PRODUCTIVITY & EXCHANGE

ACTIVITIES	OBJECTIVES				

	Build a pan-Canadian tobacco control research network	Build mechanisms for knowledge exchange and impact	Create a process to draw investigators and institutions to fill expertise gaps in the research program and strategically build research capacity	Support a training platform for mutual learning among researchers, trainees, and decision makers	Improve access to data by creating a national data repository for tobacco-related data

Annual ICE Investigator Meeting	X				

Annual Symposium	X		X	X	

Liaising with other ICE teams	X				

ICE Seed Grant	X		X		

Newsletter		X	X	X	

Learning Opportunities Program			X	X	

Summer Learning Forum	X	X	X	X	

Promotion of key awards/grants			X	X	

Data Repository	X				X

Investigator Tools (e.g., Methods Primer)		X			X

Additional Products (e.g., Literature Review, New Investigators Guide)		X			

ICE Website	X	X	X	X	X

**Table 2:** ICE Project Objectives, Outcomes and Sample Indicators

OBJECTIVE	SAMPLE OUTCOMES	SAMPLE INDICATORS
Build a pan-Canadian tobacco control research network	Increase interaction & engagement of tobacco control researchers to increase research capacity & social capital	Perception of engagement and research capacity enhancement Use of ICE-PRN resources and participation in activities (e.g., website, seed grants, newsletter, learning opportunities program)
	
	Develop equitable capacity within and across Canada	Number of provinces/territories engaged in network Number of cross-provincial seed grant teams Number and location of learning opportunity exchanges

Build mechanisms for knowledge exchange and impact	Increase personal productivity of tobacco control researchers	ICE-PRN related grant proposals, publications and presentations
	
	Develop & support connections with policy makers	Perceived effectiveness of connections Extent, pattern & depth of interaction between network members

Create a process to draw investigators and institutions to fill expertise gaps in the research program and strategically build research capacity	Increase number and breadth of researchers, trainees in tobacco control research	Number of additional universities/institutional affiliations Number of new investigator/trainee affiliations Breadth of perspectives (i.e., disciplines, sectors and geographic regions represented)

Support a training platform for mutual learning exchange and productivity among researchers, trainees, and decision makers	Participation in and perceived value of ICE-PRN activities	Number of participants in ICE-PRN activities Perceived knowledge and skills acquired as a result of participation

Improve access to data by creating a national data repository for tobacco-related data	Create data repository to help store and share tobacco control data	Number of data sets available Number of investigators/researchers who deposit data Number of peer reviewed papers/theses published based on data from data repository

We intended for these three broad foci and accompanying activities to work synergistically to build research capacity. For example, the availability of productivity tools and social networks also aided in recruitment of research community members with various disciplinary backgrounds and, by extension, the availability of certain technical tools and supports to enhance social capital.

### Strategic Recruitment

Our recruitment efforts had multiple objectives. First was to foster the development of trainees already involved in tobacco control and engage more new graduate students and post doctoral fellows to undertake programs of study and research related to individual and population level interventions for tobacco control. In this respect, the ICE-PRN partnered with the two CIHR training programs in tobacco control by co-sponsoring (with other stakeholders) an annual symposium highlighting tobacco control research as well as alerting prospective students and post doctoral fellows to potential personnel awards and training opportunities. The potential benefits of such training programs have been described elsewhere [19]. However, as previously discussed, simply adding to the roster of investigators was not deemed to be sufficient. Investigators from new or under-represented disciplinary backgrounds would bring new and different knowledge to tobacco control. This would enhance the diversity of the community and increase communication and the strength of knowledge exchange across sectors and disciplines. Therefore, a second objective of our recruitment campaign was to increase the disciplinary breadth of researchers involved in applied tobacco control. Another important consideration was the geographic distribution of research capacity. At the beginning of the project Canadian expertise tended to be concentrated in four or five institutions located in the three largest provinces (Ontario, Quebec and British Columbia). Supporting capacity building in the smaller provinces (e.g., Prince Edward Island, Saskatchewan, etc.) was not only important for ensuring that their decision makers were not disadvantaged through a lack of locally generated knowledge, but also because smaller provinces were often more willing and able to integrate research, policy and practice. Hence, a third objective of our recruitment efforts was to reduce disparities in research capacity across geographic regions of Canada, especially the provinces in the Atlantic and Prairie regions. For reasons described earlier, the final recruitment-related objective was to engage more policy makers and program providers into the research community.

The success of our recruitment efforts was evaluated using indicators such as increases in: the number of researchers and trainees involved in research immediately relevant to the tobacco control policies, practices, or future programs of research; disciplinary diversity of tobacco control researchers and trainees; and geographic distribution of tobacco control researchers, especially in the Atlantic and Prairie Provinces.

### Social Capital

Specific objectives for the project included enhancing connectivity between research community members (i.e., the frequency and depth of contact), enhancing the level of trust and cohesion among community members; the production of shared norms and priorities within the community; and the development of shared language, standards, or products.

The success of our efforts to build social capital was evaluated using indicators such as increases in: the number of multi-disciplinary and/or multi-sectoral tobacco control research teams; the level of trust between team members and members of the tobacco control research community; reciprocity between members of the broader tobacco control research community; the frequency of contacts between members of the research community and key decision makers and stakeholders outside the community; the number of collaborative products produced including policy briefs, technical papers, and journal articles, and the development of common language and standards for understanding tobacco control research, practice and policy (e.g., common definitions of key variables, minimum data sets, etc.).

### Research Productivity and Exchange

Creating social capital through social organization (i.e., structure of the research community and its linkages) was only one way to improve individual and collective capacity. A second, related method was to use technology and other tools to improve the productivity of researchers and enhance the exchange between research producers and users. Therefore, our final mechanism for enhancing capacity was to create sustainable tools (e.g., a data repository) and physical assets (as opposed to social assets or capital) that enhanced the productivity of research teams, the integration of research knowledge with practice/policy, and the integration of practice and policy problems with research programs. These last two elements may collectively be called "research exchange."

The success of productivity and exchange efforts have been evaluated using indicators such as increases in: website hits, newsletter circulation, the number of learning opportunities, and the number of products and presentations posted and accessed on the website.

## Project Team and Organization

The ICE-PRN project was conceived in 2003 by 17 researchers from seven universities and research institutions across four Canadian provinces. Founding investigators were organized into a Management Committee and four theme groups. The Management Committee composed of the PI and leaders from each of the four theme groups provided overall direction, planning and coordination for the project. The Management Committee drafted overall strategic directions, planned investigator meetings, ensured coordination of activities across the theme groups, and recommended major budget and program decisions to the full investigator team. Investigators volunteered for one or more theme groups based on their interests, as well as the desire to balance disciplinary perspectives, geographic representation, and career stage. In an effort to build leadership, a number of graduate students and early career investigators were members of theme groups. Three of the theme groups corresponded to the three mechanisms being used to generate research capacity. They had primary responsibility for planning and reviewing the project activities that were principally related to their objectives. A fourth theme group was charged with designing and implementing an evaluation strategy for the project.

## Project Activities

The ICE-PRN developed several activities and programs to help us achieve our objectives. A single activity or program often addressed more than one objective. Table 1 provides a summary of how activities related to the various objectives and mechanisms.

### Annual Symposium

One of our most significant investments was to join with several other public and non-profit organizations to plan and sponsor an annual invitational research symposium to inform tobacco control in Canada. The two day event brought together trainees, established investigators, and new investigators from a broad mix of disciplines and geographic regions. Symposium activities were designed to increase awareness of existing Canadian research (from genetic and molecular science to population level intervention), facilitate multi-disciplinary and multi-sectoral interaction, introduce prospective mentors and trainees, identify research gaps and priorities, as well as raise awareness of funding opportunities and other resources. Three symposia were funded. Over that time attendance doubled to 220 members of the Canadian tobacco control research community in attendance at the final Canadian based symposium.

### Seed grants

In order to facilitate networking and multidisciplinary team building our project provided $5000 seed grants for teams made up of an interdisciplinary mix of trainees, experienced research investigators, and decision makers (practitioners or policy developers). The seed grant program was an effective mechanism to increase knowledge exchange and mutual learning among tobacco control professionals. It also served as a catalyst for small teams to make the transition from pilot research to full-scale research investigations. Twenty teams involving 106 unique individuals (some participated in multiple teams) were funded.

### Summer Learning Forum (SLF)

Another venture was to stimulate research exchange within specific regions of Canada that previously had limited tobacco control research capacity (i.e., Atlantic Provinces; Prairie Provinces, Northern Territories). At each of the three sponsored SLFs, between 30 and 50 participants had an opportunity to interact with one another, learn about various research-related initiatives and expertise across the region, and how to gain access to resources and expertise within and beyond the region. At each forum, participants collectively identified a limited number of priority action items to create research capacity for tobacco control in the region and beyond. Seed grants (see above) were made available to teams that wished to pursue specific action steps (e.g., write a grant, write a book or paper, host a meeting, etc.).

### Population Health Data Repository

The Population Health Data Repository (PHDR) was established to hold data sets and metadata from original research conducted by tobacco control researchers. Metadata was accessible via the internet by persons affiliated with the ICE-PRN. Ownership of data in the PHDR was retained by the researchers who contributed the data sets. Persons who wished to gain access to specific data sets or sub-sets of variables completed a brief online application that described how they intended to use the data. Data was not released for secondary analysis without permission from the data owner and proof of ethics clearance from the applicant's home institution. If approved, data and associated documentation (e.g., code books, etc.) were provided to the applicant. During its first three years of operation the PHDR collected over 40 proprietary data sets and received a dozen requests for data.

### Learning Opportunities Program

This initiative assisted Canadian researchers, graduate students, research staff, policy analysts, and program providers to develop specialized skills and experience by visiting and learning from people in the research community. The ICE-PRN provided applicants with up to $1,500 per person or $5,000 per group to visit a colleague to learn new lab or analytical techniques; receive professional development and mentorship; gain experience applying research within a policy, practice or advocacy setting; or develop specific research and evaluation skills from an academic mentor. Eleven exchanges were funded over four years.

### Website

The ICE website  offered a web presence for affiliates of the ICE-PRN to post and obtain research products (including assessment and measurement tools, interventions, presentations, literature reviews, and other manuscripts). The website contained a number of aids for preparing research grants, presentations, reports and manuscripts. Job opportunities were also posted for trainees. Methodologists within the ICE-PRN prepared primers to aid students and novice investigators on how to design complex studies, select variables, and analyze complex data sets (e.g., cluster randomized designs, hierarchical designs, etc.) and deal with methodological challenges such as missing data or loss to follow-up. The website was also set up as a directory of individuals who chose to identify themselves as members of the ICE-PRN community.

### Newsletter

An electronic newsletter provided affiliates with news updates, conference announcements, feature articles about tobacco control research, and upcoming opportunities. The work of both trainees and established investigators was profiled. Thirteen issues have been circulated to a distribution list that grew to over 400 tobacco control professionals.

## Discussion and Evaluation

As the number and commitment of affiliates in our 'community' grew it enabled us to develop more activities. After nearly five years the project had co-sponsored three national symposia, catalyzed twenty new multi-disciplinary teams supported by seed grants, developed the data repository and website, hosted three Summer Learning Forums, disseminated thirteen issues of the newsletter, funded several learning opportunities, and produced a variety of manuscripts and presentations. Nearly 400 individuals participated in at least one of our activities, and many participated in multiple events. By comparison, when we began our project we estimated that less than 75 people were seriously dedicated to tobacco research in Canada, most of who were in three provinces. Initial progress was slow, but gathered momentum as program initiatives attracted new affiliates, created opportunities for them to meet and develop familiarity with one another, learn each other's language and traditions, and mutually negotiate joint activities. We learned that as valuable as single activities may be, particular combinations of activities such as newsletters, a national symposium plus seed grants or regional learning forums and learning exchange grants are synergistic in their effects.

Results of our efforts to evaluate the overall project and individual activities will be detailed in separate papers. However, overall results indicate that we achieved most of our objectives including a significant increase in the number and diversity (disciplinary background, geographic distribution, and years of experience) of investigators and trainees engaged in tobacco control research in Canada. The amount of contact and understanding between researchers and program providers and policy developers increased. More importantly, the level of engagement and trust between members of the network also increased. Network members shared data, expertise and opportunities. As a country, Canada's research productivity in tobacco control has improved. Since the first year of the project, the number of papers published in peer reviewed journals has risen steadily. There has also been a steady increase in multi-authored publications (a potential indicator of collaboration). Indeed, a recent independent international analysis shows Canada is now a global leader in the production of tobacco related research. Two of the top 10 producers are part of our network. Moreover, our investigators are broadly networked with colleagues around the world [6].

The project wasn't without its challenges. When we began there was little literature or models on how to build research capacity. Indeed, basic definitions of concepts such as "research capacity" did not exist. We hope this paper helps fill this gap. A second challenge related to understanding and providing tailored 'services' or resources to the various members of our research community. Each member was unique and had different needs from the research community. For example, a policy brief may have been required by research users. On the other hand, academic researchers required peer reviewed grants and publications to enable him/her to continue and prosper. Focusing our efforts in only one of these two arenas (i.e. providing purely policy- and program-driven tools versus a focus on purely academic outputs) would not have been sufficient to engage either research producers or users. The project needed to achieve a healthy balance in this respect in order to maintain participation from all individuals in the research community while also providing a broad spectrum of aids and tools for each 'type' of research community member.

A third challenge was to isolate the marginal benefit of our project's efforts. We continue to struggle with how to evaluate our net value or effect on the tobacco control research community after accounting for the benefit of other initiatives within Canadian tobacco control, such as the CIHR training programs discussed near the beginning of this paper. Many of our Community members were affiliated with more than one organization. Indeed, we encouraged these cross connections as a means of building capacity.

## Conclusion

The purpose of this paper was to describe an approach to capacity building in tobacco control through the creation and fostering of a Pan-Canadian Resource Network for Research, Policy and Practice for Tobacco Control. Process indicators were positive and consistent with the notion that the project facilitated more researcher involvement, greater productivity, and improved alignment between tobacco control research and practice. Notwithstanding the need for additional outcome data, the project provides a framework for building tobacco control research and decision making capacity in other countries. It demonstrates how nations can build upon the success established nearly fifty years ago with the release of reports by the Royal College of Physicians in the UK and the US Surgeon General and enhance their own capacity to adapt and extend evidence-based tobacco control.

## Competing interests

The authors declare that they have no competing interests.

## Authors' contributions

PM conceived of the project, is the Principal Investigator, and drafted the manuscript. SV and CN are Co-Investigators with the project and were involved in developing and refining the conceptual framework and related evaluation components and reviewing drafts of manuscripts. SL is a Co-Investigator with the project and is involved with activities with strategic training and recruitment. SR and MAJ are the former and current project managers and coordinate development and implementation of all network activities. All authors read and approved the final manuscript.

## References

[B1] Royal College of Physicians (1962). Smoking and Health.

[B2] Surgeon General's Advisory Committee on Smoking and Health (1964). Smoking and Health. Public Health Service Publication No 1103.

[B3] World Health Organization (2004). WHO Framework Convention on Tobacco Control.

[B4] Bal DG, Lloyd JC, Roeseler A, Shimizu R (2001). California as a model. Journal of Clinical Oncology.

[B5] Manske SR, Miller S, Moyer C, Phaneuf MR, Cameron RC (2004). Best practice in group-based smoking cessation: Results of a literature review applying effectiveness, plausibility and practicality criteria. American Journal of Health Promotion.

[B6] Kusma B, Scutaru C, Quarcoo D, Welte T, Fischer TC, Groneberg-Kloft B (2009). Tobacco control: Visualisation of research activity using density-equalizing mapping and scientometric benchmarking procedures. Int J Environ Res Public Health.

[B7] Green LW (2006). Public health asks of system science: to advance our evidence-based practice, can you help us get more practice-based evidence. American Journal of Public Health.

[B8] President's Cancer Panel (2007). 2006–2007 Annual Report: Promoting healthy lifestyles: Policy, program and personal recommendations for reducing cancer risk. http://deainfo.nci.nih.gov/ADVISORY/pcp/pcp07rpt/pcp07rpt.pdf.

[B9] Centers for Disease Control and Prevention (2007). Best Practices for Comprehensive Tobacco Control Programs – 2007. http://www.cdc.gov/tobacco/tobacco_control_programs/stateandcommunity/best_practices/pdfs/2007/BestPractices_Complete.pdf.

[B10] Wenger E (1998). Communities of Practice: Learning, Meaning, and Identity.

[B11] Kawachi I, Kennedy BP (2002). The Health of Nations: Why Inequality is Harmful to Your Health.

[B12] Lang JC (2004). Social context and social capital as enablers of knowledge intergration. Journal of Knowledge Management.

[B13] Daniel B, Schwier RA, McCalla G (2003). Social capital in virtual learning communities and distributed communities of practice. Canadian Journal of Learning and Technology.

[B14] Maibach EW, Van Duyn MAS, Bloodgood B (2006). A marketing perspective on disseminating evidence-based approaches to disease prevention and health promotion. Preventing Chronic Disease: Public Health Research, Practice and Policy.

[B15] Goering P, Butterill D, Jacobson N, Sturtevant D (2003). Linkage and exchange at the organizational level: a model of collaboration between research and policy. J Health Serv Res Policy.

[B16] Ross S, Lavis J, Rodriguez C, Woodside J, Denis JL (2003). Partnership experiences: involving decision-makers in the research process. J Health Serv Res Policy.

[B17] Kottke TE, Solberg LI, Nelson AF, Belcher DW, Caplan W, Green LW, Lydick E, Magid DJ, Rolnick SJ, Woolf SH (2008). Optimizing Practice Through Research: A New Perspective to Solve an Old Problem. Ann Fam Med.

[B18] Green LW (2001). From research to best practices in other settings and populations. American Journal of Health Behaviour.

[B19] Leatherdale ST, Viehbeck S, Murphy C, Norman C, Schultz A (2007). The tobacco control community of tomorrow: A vision for training. The Canadian Journal of Public Health.

